# Effect of Extrusion or Fermentation on Physicochemical and Digestive Properties of Barley Powder

**DOI:** 10.3389/fnut.2021.794355

**Published:** 2022-02-10

**Authors:** Xiang Xiao, Jiaying Li, Hao Xiong, Wenxuan Tui, Ying Zhu, Jiayan Zhang

**Affiliations:** ^1^School of Food and Biological Engineering, Jiangsu University, Zhenjiang, China; ^2^State Key Laboratory of Food Science and Technology, Jiangnan University, Wuxi, China

**Keywords:** extrusion, fermentation, rheological properties, digestive properties, antioxidant activity

## Abstract

In this work, the effect of extrusion and fermentation on the physicochemical and digestive properties of barley powder was studied. The results showed that the contents of phenolics, β-glucan, protein, and lipid decreased after extrusion. The contents of nutrients (except lipid) increased after fermentation. Both extrusion and fermentation of barley can lead to the darkening of the color and effectively optimize the palatability by reducing the viscosity. *In vitro* digestion of starch showed that the content of as rapidly digestible starch increased after extrusion and fermentation. The contents of ferulic acid, 2-hydroxybenzoic acid, and caffeic acid decreased after extrusion, while the contents of chlorogenic acid, p-coumaric acid, and ferulic acid increased after fermentation. Basically, the content of all the phenolic showed an increasing trend after digestion. The antioxidant activity decreased after extrusion and increased after fermentation. Therefore, the nutritional composition and properties of barley powder were changed under the two processing methods.

## Introduction

In recent years, metabolic syndrome, represented by obesity, hyperglycemia, hyperlipidemia, and diabetes mellitus, has become one of the most important non-communicable chronic diseases affecting human health. Barley is a new type of health food, whose overall nutritional index meets the requirements of high plant protein, high vitamin, high cellulose, low fat, and low sugar proposed by modern nutrition science. In addition, as a grain with a low glycemic index, barley can also be used to prevent chronic diseases such as diabetes and obesity (1).

Extrusion can greatly improve the digestibility of nutrients. Due to the short duration of the processing of food raw materials, the harmful effects of browning and nutritional damage on the nutrients of the raw materials are reduced to a large extent. Extrusion is a common processing method in the food industry, and it has the advantages of high production efficiency, simple process, low energy consumption, and low cost.

Fermentation is an effective method to improve the quality of barley. *Lactobacillus plantarum* is a common probiotic in food and it can regulate human cholesterol, blood pressure, and intestinal microorganism. Fermentation of barley using *Lactobacillus plantarum* can improve the nutritional value of barley and also can reduce the content of phytic acid. Therefore, the combination of *Lactobacillus plantarum* fermentation with barley is beneficial to improve its functional active components and enhance its physiological and nutritional functions (2).

This paper mainly studied the effects of extrusion and fermentation treatments on the nutrient composition, rheological, and digestive properties of barley powder, and analyzed the advantages and disadvantages of these changes.

## Materials and Methods

### Materials and Reagents

Yangzhou's No. 3 Barley Powder (BP) was purchased from Yancheng Shuangzeng Agrochemical Technology Co. LTD (Yancheng, China). Extruded Barley Powder (EBP, 40 Hz of screw rotation, 30 Hz of feeding speed, 140°C of sleeve temperature, and 30% of raw water content) and fermented barley powder (FBP, fermentation with *Lactobacillus plantarum* dy-1) were prepared in our laboratory.

α-amylase, glucosidase, pepsin, trypsin, and bile salt were purchased from Sigma Company (St. Louis, MO, United States) as biological reagents. Gallic acid, catechin, epicatechin, chlorogenic acid, ferulic acid, caffeic acid, 2-hydroxybenzoic acid, p-coumaric acid, vanillin acid, and 3, 4-dihydroxybenzoic acid were purchased from Tixi Ai (Shanghai) Chemical Industrial Development Co, Ltd.

### Determination of Nutritional Components

#### Determination of Total Phenolics

The total phenolic content was determined by the method described by Rani et al. (3) with some modifications. Briefly, 1 ml of sample extract was diluted with 5 ml water and 3 ml of Na_2_CO_3_ (7.5%) was added. Subsequently, the test tube was kept for 90 min incubation in the 45°C water bath and absorbance was measured at 750 nm. A Gallic acid standard curve was made to determine the phenolic content.

#### Determination of β-Glucan

β-Glucan content was determined by the method described by 4 with some modifications. Briefly, 0.5 ml of sample extract was diluted with 1.5 ml water and 4 ml of Congo red solution was added. Subsequently, the test tube was kept for 10 min incubation in the 20°C water bath and absorbance was measured at 550 nm. A β-glucan standard curve was made to determine the β-glucan content.

#### Determination of Protein Content

The total nitrogen content was estimated by an azotometer (Milan, Italy). The protein content was calculated with the conversion factor of 6.25.

#### Determination of Fat Content

The fat content was determined by following the AOAC (2006) standard method number 2003.05.

#### Determination of Free Amino Acid Content

The content of free amino acids was determined by the amino acid analyzer according to the method of Hu et al. (5). The results were expressed as the percentage of each amino acid in the total amino acids with the exception of tryptophan.

### Dissolving Characteristics and Rheological Characteristics

#### Determination of the Color

The colors of barley samples were determined using the HunterLab UltraScan Pro according to the method described by Morris et al. (6) with some modifications. The L^*^ value represents the lightness, 0–100 represents dark to light. The a^*^ value represents the degree of the red-green and the b^*^ value represents the degree of the yellow-blue.

#### Determination of WSI and WAI

The water solubility index (WSI) and water absorption index (WAI) were measured by the method of Haoqiang Wang et al. (7), with some modifications. A total of 2.5 g of samples were dispersed in 30 ml distilled water and shaken in the water bath at 30°C for 30 min, then centrifuged at 7000 × g for 15 min. The weight of the sediment was taken as WAI and was expressed as the unit g/g. The WSI is the weight of dry solids in the supernatant, which is expressed as a percentage of the original weight of the sample.

#### Determination of Pasting Properties

Pasting properties were determined by the method described by H. Wang et al. (8) with some modifications. Briefly, the pasting properties of samples were measured by a rapid visco-analyzer (RVA) combined with the Thermocline for Windows (TCW_3_) software. The basic weight of the sample was 3 g (on a raw basis) and was automatically corrected to a moisture basis of 14%. Approximately, 3.0 g of samples and 25 ml of distilled water were directly added into an RVA canister. Then, the slurry was homogenized manually by the plastic paddle to avert lumps formation prior to the RVA run. The canister with the sample and paddle was inserted into the RVA Instrument and the analysis was started. The samples were heated from 50 to 95°C with a heating rate of 12°C/min, maintained at 95°C for 3.5 min, and then cooled to 50°C at the same rate and maintained at 50°C for 5 min. The following characteristics were then evaluated: peak viscosity, trough viscosity, final viscosity, breakdown, setback, peak time, and pasting temperature.

#### Static Viscoelasticity Analysis

The efficacy of barley in lowering blood lipid and cholesterol is closely related to its viscosity. The rheological property of the sample can be further analyzed by measuring the static viscoelasticity. Viscoelastic measurements were performed using a TA rheometer equipped with parallel plate geometry (40 mm). Firstly, 0.6 g sample was dissolved in 10 ml distilled water and mixed to prepare the solution with 6% concentration. After the fixture was installed and the calibration was carried out, about 1.5 ml sample solution was taken and dropped in the center of the plate. A frequency sweep test for starch gels was performed at 25°C from 0.01 to 1,000 s^−1^ within the linear viscoelastic range.

### *In vitro* Starch Digestion

The digestibility of starch was determined according to the procedure, as follows. First, 0.2 M sodium acetate buffer (pH 6.0 containing 200 mM calcium chloride, 0.49 mM magnesium chloride, and 0.02% sodium azide) was prepared and stored in a refrigerator at 4°C.

The *in vitro* starch digestion test: Each sample (50 mg) was suspended in a 50 ml centrifuge tube containing 2 ml deionized water, and then mixed well. Then, 8.0 ml of enzyme solution (37°C) was added to the tube, followed by incubation in a water bath at 37°C with shaking, at a stirring speed of 300 rpm. After the time periods of 0, 10, 20, 60, 120, and 180 min, the tube was shaken and 0.1 ml aliquot was quickly taken and transferred to a 1.5 ml centrifuge tube containing 0.9 ml absolute ethanol to deactivate the enzymes. The tube was then returned for further time course digestion.

Starch digestibility was calculated using the GOPOD method by determining the glucose content. Based on the hydrolysis rate, starch was defined as rapidly digestible starch (RDS), slowly digestible starch (SDS), and resistant starch (RS).

### *In vitro* Digestion of Phenolic Compounds

#### Extraction of Phenolic Compounds

The extraction of polyphenolic compounds was performed according to the protocol of Horax et al. (9) with modifications. A total of 10 ml of a methanol solution was added to 1 g of each sample and stirred for 2 h at 200 rpm, then treated for 15 min in an ultrasonic bath and finally centrifuged for 10 min at 6,000 rpm. The pellet obtained was subjected to a second extraction and resuspended in 10 ml of methanol solution and the second extraction was performed as described before.

#### *In vitro* Digestion

A static model that simulated gastrointestinal digestion was developed, following the method described by Minekus et al. (10). The detailed composition of simulated salivary fluid (SSF), simulated gastric fluid (SGF), and simulated intestinal fluid (SIF) is given in Table 1, and they were prepared from stock solutions fresh daily and kept at 37°C before use.

**Table 1:** Preparation of stock solution of simulated digestion fluids.

	**Concentration (M)**	**SSF (mM)**	**SGF (mM)**	**SIF (mM)**
KCl	0.50	18.88	8.63	8.50
KH_2_PO_4_	0.50	4.63	1.13	1.00
NaHCO_3_	1.00	17.00	31.25	106.25
NaCl	2.00	-	59.00	48.00
MgCl_2_(H_2_O)_6_	0.15	0.19	0.13	0.41
(NH_4_)_2_CO_3_	0.50	0.08	0.63	-

#### *In vitro* Simulation of Saliva Digestion (SD)

A total of 4 ml SSF was added to a 5.0 g barley powder sample and mixed well. Then, 15 mg α-amylase, 25 μL of CaCl_2_ solution, and 975 μL water was added and shaken at 37°C for 2 min away from light.

#### *In vitro* Simulation of Gastric Digestion (GD)

A total of 160 mg pepsin (250 U/mg) was added to 8.0 ml SGF, and it is added to saliva digestive products. Then, 5 μL CaCl_2_ solution and 1990 μL water were added. Thew pH was adjusted to 3 with HCl, mixed well, and shaken away from light for 2 h.

#### *In vitro* Simulation of Intestinal Digestion (ID)

About 144 mg bile salt was added to 16.0 ml SIF and stirred at 37°C for 20–30 min. Then, 535 mg trypsinase and 40 μL CaCl_2_ solution were added to the gastric digestible products. The pH was adjusted to 7 with NaOH solution. After simulation, the samples were frozen at −20°C, lyophilized, and submitted to extraction as described above (Section Extraction of Phenolic Compounds).

#### Determination of Phenolic Composition

The individual phenolic compounds in the above extracts were quantified using a Shimazu LC-20AD HPLC system. A ZORBAX SB-Aq column (5 m, 4.6 mm × 250 mm ID, Agilent) was used at a column temperature of 30°C. The mobile phase consisted of a 0.1% aqueous solution of formic acid (solution A) and acetonitrile (solution B) with the following gradient program: 0–10 min, solution B 3–3%; 10–20 min, solution B 3–80%; 20–30 min, solution B 80–3%; 30–35 min, solution B 3–3%. Other chromatographic conditions included a constant flow rate of 0.8 ml/min, an injection volume of 20 μL, a run time of 35 min, and a detection wavelength of 254 and 320 nm. Prior to analysis, all samples were filtered through a 0.25–l m membrane filter. The identification of each peak was based on the retention time and the chromatography of authentic standards. The concentrations of each compound were calculated according to a standard curve, and the results were expressed as μg/g DW of barley powder.

### Determination of Antioxidant Activity

#### Determination of DPPH Radical Scavenging Activity

The method for determination of DPPH radical scavenging ability is based on measurement of the loss of DPPH radical color at 517 nm after reaction with sample extract and was performed as described by Brand–Williams et al. (11). Sample extracts (2 ml) were mixed with 2 ml of DPPH radical solution, incubating for 30 min in the dark and measuring absorbance at 517 nm. Sample extracts and anhydrous ethanol were used as a control group. Anhydrous ethanol and DPPH radical solution were used as a blank group. Results were expressed as the percentage of free radical scavenging.

#### Determination of ABTS Radical Cation Scavenging Activity

2, 2′ -azino-BIS (3-ethylbenzothiazoline-6-sulfonic acid) (ABTS) radical cation scavenging capacity was measured using the modified method described by Sun et al. (12). Diluted the absorbance of ABTS radical cation solution to 0.70 ± 0.02 at 734 nm. Extracts (0.1 ml) were mixed with 2 ml ABTS radical cation, incubating 2–6 min, and measuring the absorbance at 734 nm. Sample extracts and anhydrous ethanol were used as a control group. Anhydrous ethanol and ABTS radical cation were used as the blank group. Results were expressed as the percentage of free radical scavenging.

#### Determination of Ferric Reducing Antioxidant Power-FRAP Method

Ferric Reducing Antioxidant Power (FRAP) assay was performed according to a modified method reported by Benzie and Strain (13). Fresh FRAP working reagent was prepared by mixing 25 ml 300 mM acetate buffer, 2.5 ml TPTZ solution, and 2.5 ml of 20 mM FeCl_3_•6H_2_O solution. FeSO_4_ solution was used as the standard solution. A total of 180 μL FRAP solution was mixed with the standard solution in different concentrations and sample extracts, respectively. After incubation at 37°C for 3–5 min, the absorbance was detected at 593 nm.

### Statistical Analysis

Each experiment was carried out in triplicate. Experimental design and data analysis were performed with IBM SPSS statistical software. Data were analyzed by ANOVA and expressed as mean ± SD. *P* < 0.05 was considered to be statistically significant.

## Results and Discussion

### Nutritional Components Analysis

#### The Content of Free Phenol, β-Glucan, Protein, and Fat

To evaluate the effects of extrusion and fermentation of barley powder, changes in main nutrients and amino acid contents were determined, as shown in Table 2. Compared with BP, the content of free phenol, β-glucan, and fat in EBP were all decreased by 42.33, 8.09, and 59%, respectively. The content of free phenol and β-glucan in FBP was increased by 35.47 and 212.53%, the content of fat in FBP was decreased by 70.88%. The phenol and β-glucan content of FBP was significantly higher than that of BP and EBP, whereas the content of fat was lower than that in EBP, so FBP has greater significance in a healthy diet. The decrease of fat content and increase of β-glucan content in cereal can reduce its GI (glycemic index), which can be used in healthy food to reduce cholesterol, blood sugar, and blood fat of the human body.

**Table 2:** Main nutritional and function component of BP, EBP, and FBP.

	**Free phenol (mg/g)**	**β-glucan (%)**	**Protein (%)**	**Fat (%)**
BP	25.63 ± 1.15^b^	3.83 ± 0.68^b^	13.99 ± 0.32^a^	2.61 ± 0.21^a^
EBP	14.78 ± 0.52^c^	3.52 ± 0.17^b^	13.50 ± 1.09^a^	1.07 ± 0.21^b^
FBP	34.72 ± 0.61^a^	11.97 ± 1.42^a^	14.67 ± 0.41^a^	0.76 ± 0.21^c^

a,b,c *Represents the highest figure in the data*.

The phenolic compounds are decomposed by high temperatures and the complex polyphenols are decomposed into other phenolic or non-phenolic compounds during extrusion, therefore, there is a loss of free phenol content. It is reported that there is a significant loss of total phenolics during the extrusion of oat cereals (14). These reports corroborate well with the findings of the present study. The increase of free phenol content after fermentation was mainly due to the acid hydrolysis produced by *Lactobacillus* fermentation that led to the release of the bound compound into a free state. Similarly, it found that the lactic acid bacteria fermentation increased the contents of the free, soluble conjugate, and total phenolics by 90.7, 17.2, and 37.1% respectively (15). Mao et al. (16) found that the total phenol content was increased significantly after fermentation, which was 39.10% higher than the raw wheat bran.

Tosh et al. (17) found that the depolymerization of the cell wall caused by pressure during the extrusion process leads to the loss of integrity, resulting in a decrease in the content of β-glucan in cereals. Similarly, it has been reported that it exhibited a significant decrease of 8% in β-glucan content when the extrusion temperature was constant at 150°C, and when extrusion was carried out at 170°C, β-glucan content lowered by 10% (18). The apparent increase in soluble β-glucan observed after fermentation might be due to enhanced activity of enzymes like β-glucanases and carboxypeptidases which cause degradation of total and insoluble β-glucan content into soluble β-glucan (4).

The decrease of the fat content of EBP might be due to the formation of lipid complexes under high temperatures and pressures. Hagenimana et al. (19) reported that decreases in the fat, protein, and reducing sugar content occurred through the many chemical and structural transformations such as starch gelatinization, protein denaturation, and complex formation between amylose and lipids during the extrusion process. Similarly, the fat content of white and red Ginseng samples significantly decreased from 1.29 to 0.23% after extrusion (20). The apparent decrease of fat content in FBP may be due to the degradation of lipids by lipase during fermentation, which is in agreement with what has been reported by Al-Ansi et al. (21). Besides, it is reported that the endo amylase played a vital role in starch hydrolysis during the fermentation process (22). Similarly, Chai et al. (23) found that the fat content of the Rambutan seed reduced by 22% whereas its free fatty acid content increased by 4.3 folds after 10 days of fermentation.

#### The Content of Amino Acids

As shown in Table 3, the most abundant amino acids in barley are glutamine, valine, leucine, phenylalanine, and proline. The contents of most amino acids decreased after extrusions, such as glutamic acid, leucine, and arginine, which may be due to the mutual conversion and decomposition of amino acids into small molecules of ammonia under the influence of high temperature and high pressure during extrusion. Similarly, the methionine and cysteine content of peas decreased after extrusion treatment (24).

**Table 3:** Free amino acids content (%) of BP, EBP, and FBP.

	**BP**	**EBP**	**FBP**
Aspartic acid	1.10 ± 0.03^a^	0.92 ± 0.05^b^	1.05 ± 0.03^a^
Threonine	0.66 ± 0.02^a^	0.58 ± 0.02^b^	0.68 ± 0.01^a^
Serine	0.86 ± 0.01^a^	0.79 ± 0.07^b^	0.90 ± 0.01^a^
Glutamine	5.40 ± 0.13^b^	4.89 ± 0.15^c^	5.56 ± 0.21^a^
Glycine	0.78 ± 0.02^b^	0.66 ± 0.01^c^	0.95 ± 0.03^a^
Alanine	0.84 ± 0.03^b^	0.71 ± 0.03^c^	0.91 ± 0.06^a^
Cysteine	0.29 ± 0.01^c^	0.44 ± 0.01^a^	0.38 ± 0.03^b^
Valine	1.29 ± 0.08^a^	1.06 ± 0.04^b^	1.22 ± 0.05^a^
Methionine	0.24 ± 0.01^b^	0.23 ± 0.01^b^	0.27 ± 0.02^a^
Isoleucine	0.76 ± 0.04^a^	0.64 ± 0.01^b^	0.76 ± 0.03^a^
Leucine	1.57 ± 0.02^a^	1.28 ± 0.05^b^	1.55 ± 0.08^a^
Tyrosine	0.97 ± 0.03^a^	0.88 ± 0.13^b^	1.01 ± 0.11^a^
Phenylalanine	1.37 ± 0.17^a^	1.26 ± 0.13^b^	1.38 ± 0.09^a^
Lysine	0.68 ± 0.01^a^	0.55 ± 0.01^b^	0.67 ± 0.03^a^
Hlstidine	0.42 ± 0.03^b^	0.36 ± 0.01^b^	0.84 ± 0.02^a^
Argnine	0.94 ± 0.03^a^	0.79 ± 0.04^b^	0.94 ± 0.03^a^
Proline	2.38 ± 0.12^b^	2.15 ± 0.05^c^	2.52 ± 0.10^a^

a,b,c *Represents the highest figure in the data*.

The proportion of essential amino acids in FBP had increased, such as glutamic acid, glycine, alanine, and methionine. It may be due to the mutual conversion of amino acids during the fermentation of *Lactobacillus plantarum*. LAB fermentation can activate endogenous protease activity of barley powder by decreasing pH (25), thereby hydrolyzing protein and increasing free amino acid content. Additionally, proteases produced by microorganisms during fermentation could also hydrolyze the protein of wheat bran (26). Similarly, Hu et al. (5) found that the proportion of essential amino acids increased after fermentation, such as threonine, valine, and isoleucine Dallagnol. Mao et al. (16) found that methionine and cysteine of wheat bran were increased by 315 and 33%, respectively, after fermentation, the contents of tyrosine, phenylalanine, isoleucine, Val, and other essential amino acids were also significantly increased.

### Effects of Extrusion and Fermentation on Physicochemical Properties

#### Color

Color is an important sensory quality of barley powder. The brightness (L^*^), red–green (a^*^), and yellow–blue (b^*^) of the samples are shown in Table 4. In general, the L^*^ value of EBP decreases, while a^*^ and b^*^ values increase, L^*^ and b^*^ values of FBP decrease, while a^*^ value increases. The BP was brighter than the EBP, which was brighter than the FBP. The EBP was greener than the FBP, which was greener than the BP. The EBP was bluer than the BP, which was bluer than the FBP.

**Table 4:** Color of BP, EBP, and FBP.

	L*	a*	b*
BP	92.07 ± 0.02^a^	1.26 ± 0.01^c^	5.48 ± 0.04^b^
EBP	88.28 ± 0.01^b^	1.76 ± 0.03^a^	9.53 ± 0.01^a^
FBP	86.32 ± 0.02^c^	1.39 ± 0.02^b^	4.15 ± 0.05^c^

a,b,c *Represents the highest figure in the data*.

L* represents the lightness, 0–100 represents dark to light, a* represents the degree of the red-green and b* represents the degree of the yellow-blue.

The observed change in color of EBP may be attributed to the gelatinization caused by high temperature and high-pressure during extrusion. Reduced sugars and proteins (amino acids) in grain can react under high processing temperatures to promote non-enzymatic browning (Maillard reaction), which results in the darkening of the EBP (27). Similarly, previous researchers have observed that extrusion of the whey protein concentrate and corn starch resulted in higher color differences with the increase in the amylose content (28). The color change of FBP is also related to the browning reaction where a fluctuation in L^*^ values and an increase in a^*^ values indicate more browning (29). Similarly, previous researchers have observed that there were significant differences in a^*^ and b^*^ values obtained between unfermented and fermented seeds of rambutan (30).

#### WSI and WAI

The water solubility index (WSI) of barley powder is related to the degree of starch degradation. As shown in Table 5, the WSI was significantly increased from 9.73 (BP) to 10.66% (EBP) after the extrusion of barley powder. It was also found that the WSI of pea flour significantly increased after extrusion from 11.70 to 54.9% (31). It has been reported that the crystalline structure was disrupted, and starch was gelatinized during extrusion so that the amylose leached out to cause the increase in the WSI (32). The WSI of FBP was found to be 18.00%, which was remarkably higher than the BP and EBP. It has been reported that molecular components of starch are degraded during fermentation which results in increased soluble components. Similarly, the gel forming capacity of the fermented rice black gram mix flour was significantly higher than the extrudate products (3).

**Table 5:** WSI and WAI of BP, EBP, and FBP.

	**WSI (%)**	**WAI (%)**
BP	9.73 ± 0.23^c^	190.13 ± 8.89^c^
EBP	10.66 ± 0.50^a^	462.93 ± 3.80^c^
FBP	18.00 ± 0.40^c^	696.80 ± 4.50^a^

a,b,c *Represents the highest figure in the data*.

The WAI reflects the water absorption capacity of barley powder and is significantly dependent on molecular interactions in the crystalline and amorphous regions of the starch granule (33). As shown in Table 2, the WAI of EBP was 462.93% higher than BP. It was also found that the WAI of rice starch significantly increased after extrusion from 136 to 246–412% at different moisture contents (32).

#### Pasting Properties

Pasting properties are very important in the process of choosing new food manufacturing additives, such as thickeners, emulsifiers, binders, or other components related to paste viscosity (34). Therefore, the result may be used for the indirect estimation of the intensity of the extrusion and fermentation treatment of barley powder. As shown in Table 6, the PV, TV, FV, SB, PT, and PTT of BP are higher than EBP and EBP, which indicated that the BP showed more stable viscosity properties during measurements. The starch pasting levels of EBP and FBP were increased, which could effectively optimize the taste of barley products, and further facilitate the development of barley edible products.

**Table 6:** Pasting properties of BP, EBP, and FBP.

	**PV (cp)**	**TV (cp)**	**BD (cp)**	**FV (cp)**	**SB (cp)**	**PT (min)**	**PTT (**°**C)**
BP	4,731.00 ± 94.62^a^	3,314.00 ± 36.04^a^	1,417.00 ± 41.58^b^	5,224.33 ± 88.24^a^	1,910.33 ± 6.51^a^	6.73 ± 0.07^a^	72.02 ± 0.42^a^
EBP	2,189.67 ± 75.55^c^	664.67 ± 41.10^c^	1,786.00 ± 99.01^b^	1,305.00 ± 73.91^c^	677.00 ± 45.97^c^	5.33 ± 0.11^b^	66.18 ± 7.20^a^
FBP	3,230.33 ± 75.93^b^	1,005.00 ± 16.46^b^	2,225.33 ± 116.09^a^	1,822.00 ± 38.43^b^	817.00 ± 35.04^b^	1.09 ± 0.03^c^	52.60 ± 2.08^b^

a,b,c *Represents the highest figure in the data*.

The peak viscosity of samples is influenced by amylose content, starch leaching, as well as the content of other components, such as proteins, lipids, and fibers (35). The decrease in PV of EBP may be attributable to starch pasting, protein denaturation, and interactions between starch and protein (36). Additionally, the reduced paste viscosity could be attributed to the formation of compact starch granules during high temperature treatment, which had higher resistance to heat and shear during pasting. The decline in PV of FBP might be caused by amylopectin degradation, disruption of granular structures, and disordering of hierarchical structures of starch (8).

Trough viscosity (TV) indicates the sensitivity of starch granules to shearing. During the 95°C period, swollen granules disintegrated due to the shearing of the paddle and rotating bowl, resulting in a decrease in viscosity known as viscosity breakdown. Final viscosity (FV) indicates a retrogradation tendency of the soluble amylose after cooling, or the ability of the starch paste to form a gel (35). Breakdown (BD) is a characteristic feature of shear resistance and heat resistance of starch hot paste. Setback (SB) reflects the stability and aging trend of starch cold paste, a lower setback value indicates higher stability. The content and structure of Amylose and amylopectin in barley flour can affect the pasting time (PT) and pasting temperature (PTT), higher amylose content, and the longer branched chain of amylopectin cause higher heat to dissolve the crystal and higher PTT.

The decrease in TV, FV, BD, and SB of EBP may be attributable to the speed and temperature of the high extruder screw. Similar observations were reported by Siddiq et al. (37), and Mitrus et al. (38). The reduction in TV, FV, BD, and SB of FBP may be attributable to the higher α-amylase activity; the starch grains tend to lose resistance to swelling, and thus, the viscosity declines (39). Similar observations were reported that the fermentation significantly decreased the pasting properties by Al-Ansi et al. (40) and Zhao et al. (41).

#### Static Viscoelasticity

**Figure 1:**
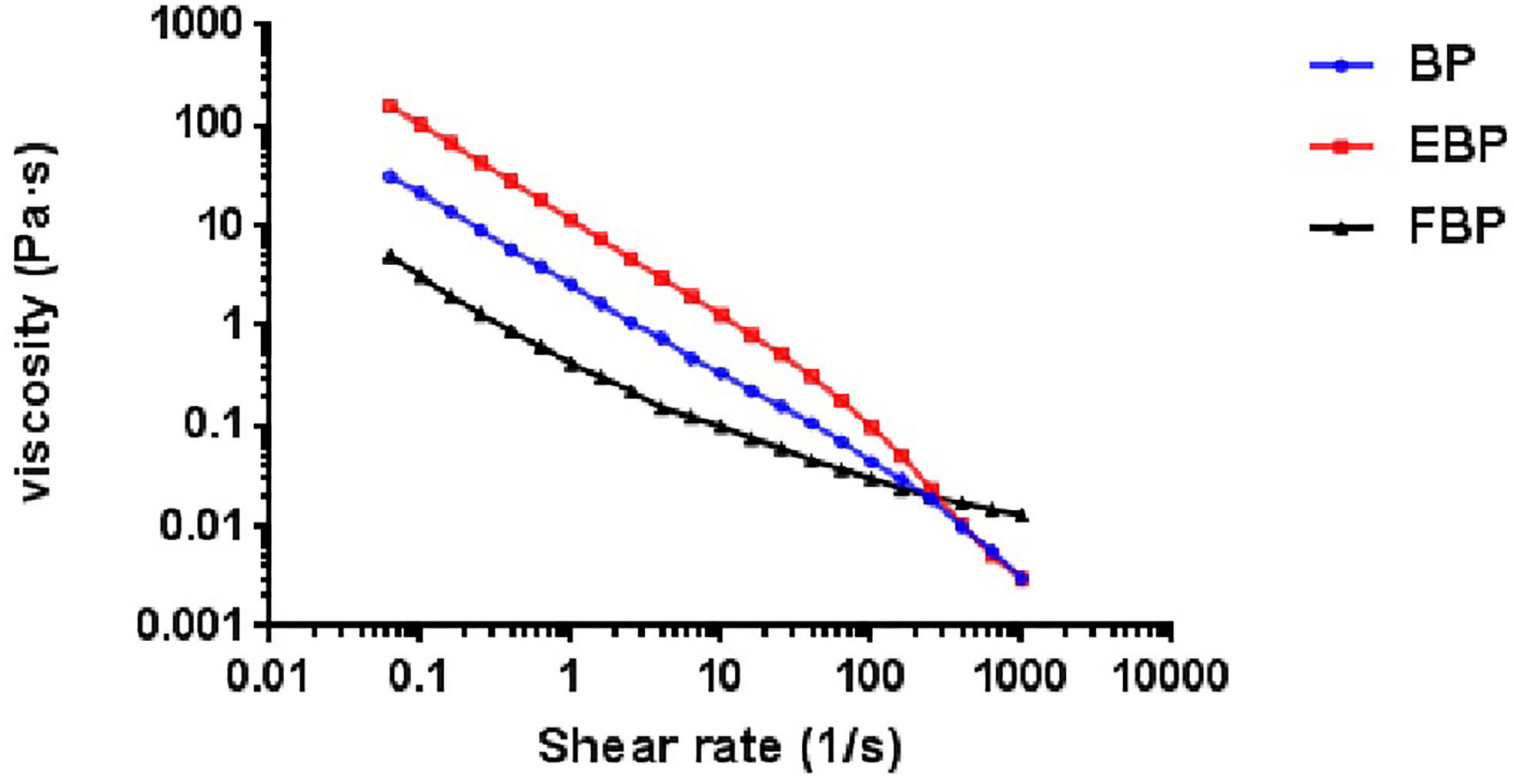
The static viscoelasticity of BP, EBP, and FBP.

As shown in Figure 1, the static rheological properties of BP, EBP, and FBP show the same trend of viscosity change, and the viscosity decreases with the increase of shear rate which might be due to the increasing external force with the increase of shear rate, and the destruction of the hydration structure between polysaccharide molecules and water molecules, leading to the decrease of viscosity. The initial viscosity of EBP is higher than that of BP, and that of FBP is lower than that of BP. The decrease of viscosity is beneficial to improve the palatability of popular products and further promote the development and utilization of barley in the food industry.

### *In vitro* Digestion of Starch

#### Starch Decomposition Rate

**Figure 2:**
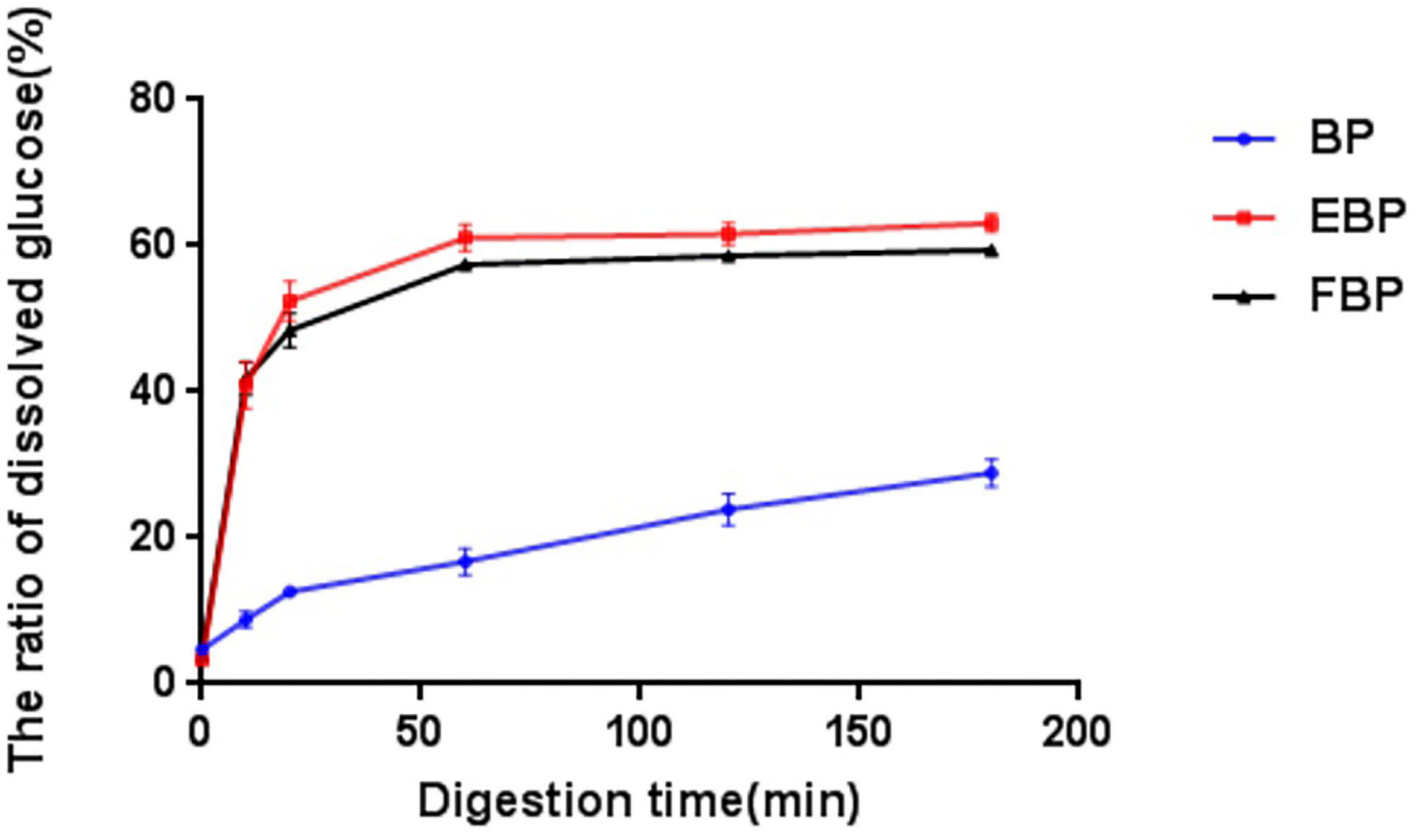
The starch decomposition rate during digestion of BP, EBP, and FBP.

As can be seen from Figure 2, during the digestion of the sample *in vitro*, the hydrolysis rate of starch increased rapidly in the first 60 min, especially in the first 20 min. The hydrolysis rate of starch from high to low was EBP, FBP, and BP at 180 min. The results showed that the digestibility of barley powder could be improved by extrusion and fermentation, and it could be rapidly decomposed into reducing sugars in the human body for absorption and utilization. Similar observations were reported by de la Rosa-Millán et al. (42).

#### Contents of TS, RDS, SDS, and RS

The contents of RDS, SDS, and RS in the BP, EBP, and FBP are shown in Table 7. Compared to the BP, EBP, and FBP had less SDS and RS, but more RDS, which indicated that both extrusion and fermentation could accelerate the digestion of the starch by transforming SDS and RS into RDS, significantly improving the digestibility of barley powder, and the effect of EBP was more obvious than that of FBP.

**Table 7:** Content of TS, RDS, SDS and RS of BP, EBP, and FBP (%).

	**RDS**	**SDS**	**RS**
BP	14.92 ± 1.49^b^	18.58 ± 0.88^a^	68.66 ± 1.69^a^
EBP	73.77 ± 2.01^a^	11.77 ± 0.76^b^	14.46 ± 1.25^c^
FBP	64.30 ± 1.16^a^	6.59 ± 0.52^b^	29.11 ± 1.16^b^

a,b,c *Represents the highest figure in the data*.

Rapidly digestible starch can be quickly digested by the human body, and its content is one of the indicators of digestible foods that can predict elevated glycaemic index. The increase in RDS of EBP could be due to the direct effect of the extrusion treatment on starch granules as BP contains little or no detectable α-amylose inhibitors (43). SDS results in the slow release of glucose into the blood; such property is advantageous for human health as it prevents diabetes and obesity. RS is known as a type of dietary fiber that cannot be digested in the small intestine. The significant increase in RDS of EBP, with a corresponding decrease in the RS, is probably due to the extrusion treatment decreasing starch crystallinity and increasing enzyme availability results in a decrease in RS content. A similar result has been reported by Chen et al. (44).

There was also a significant increase in the RDS content after fermentation from 14.92 to 64.30% and the RS content decreased from 68.66 to 29.11%. Golden found that the significant increase in RDS of FBP, with a corresponding decrease in the RS might be due to the disruption of the ordered structure of starch chains by actions of microbial organisms during fermentation, thereby increasing the accessibility of starch granules to the digestive enzymes (45). A similar result has been reported for the sorghum flour and cassava flour by Inglett et al. (46) and Elkhalifa et al. (47).

### *In vitro* Digestion of Polyphenols

#### Polyphenols Component

The 7 phenolic compounds from samples were analyzed and are presented in Table 8. According to the HPLC analyses, the total content of each phenolic compound in abstracts from barley powder significantly increased during fermentation while decreased during extrusion. Quantitative results indicated that cumaric acid was the most abundant compound in barley, followed by 2-hydroxybenzoic acid, chlorogenic acid, and 3, 4-dihydroxybenzoic acid, additionally, after *in vitro* simulation of saliva, gastric, and intestinal digestion.

**Table 8:** The content of phenolic compounds before and after digestion *in vitro*.

	**3, 4-dihydroxybenzoic acid**	**Chlorogenic acid**	**Caffeic acid**	**p-coumaric acid**	**Ferulic acid**	**2-hydroxybenzoic acid**	**Vanillic acid**	**Total**
BP (Before digestion)	12.24 ± 0.56^c^	14.67 ± 0.27^d^	7.90 ± 0.28^b^	154.72 ± 2.37^d^	12.17 ± 0.22^b^	20.35 ± 0.38^b^	6.81 ± 0.17^b^	228.86 ± 4.86^d^
EBP (Before digestion)	9.18 ± 0.83^d^	11.68 ± 0.25^e^	3.30 ± 0.26^c^	141.94 ± 2.84^d^	3.87 ± 0.69^c^	2.76 ± 0.06^c^	3.85 ± 0.37^c^	176.59 ± 6.63^e^
FBP (Before digestion)	12.36 ± 0.64^c^	17.34 ± 0.65^c^	10.61 ± 0.74^a^	214.91 ± 3.59^c^	16.19 ± 0.74^a^	25.81 ± 1.36^b^	7.22 ± 0.82^b^	304.43 ± 9.74^c^
BP (After digestion)	15.58 ± 0.74^b^	26.99 ± 0.98^b^	8.46 ± 0.50^b^	261.14 ± 1.48^b^	4.38 ± 0.56^c^	40.36 ± 1.74^a^	9.63 ± 1.48^a^	366.54 ± 7.48^b^
EBP (After digestion)	14.98 ± 0.26^b^	22.02 ± 0.42^b^	5.79 ± 0.37^c^	222.91 ± 2.94^c^	8.53 ± 0.85^b^	18.44 ± 0.59^b^	9.01 ± 0.94^a^	301.70 ± 6.06^c^
FBP (After digestion)	17.39 ± 0.47^a^	44.70 ± 2.20^a^	11.65 ± 0.99^a^	331.70 ± 3.58^a^	18.32 ± 0.59^a^	28.14 ± 0.37^b^	10.80 ± 0.42^a^	462.69 ± 9.04^a^

a,b,c *Represents the highest figure in the data*.

Compared to the BP, the content of ferulic acid, 2-hydroxybenzoic acid, and caffeic acid were significantly decreased in EBP. Extrusion could inevitably lead to a decrease of free phenolic acids because of decomposition caused by the high temperature. A similar result has been reported by Zeng et al. (48) for the extruded brown rice and extruded wheat. The contents of chlorogenic acid, p-coumaric acid, and ferulic acid were dramatically increased in FBP. It was widely reported that phenolic acids in cereals were mainly in bound form and involved in linkages of ester, ether, or acetal bonds to cellulose, proteins, or lignin. These linkages break due to the enzymes produced during fermentation such as esterases and proteases. A similar result has been reported for the fermented rice bran (15).

#### Antioxidant Activity

As shown in Table 9, the DPPH•, ABTS radical cation, and FRAP of BP are higher than those of EBP and lower than those of FBP, indicating that the antioxidant capacity of barley powder was partially lost during the extrusion process and enhanced during the fermentation process. It was observed that there was a positive correlation between the total phenolic content and antioxidant activity.

**Table 9:** Antioxidant activity of BP, EBP, and FBP (%).

	**DPPH•scavenging activity**	**ABTS•^+^ scavenging activity**	**FRAP**
BP	46.03 ± 2.32^b^	14.25 ± 0.28^b^	52.39 ± 3.21^b^
EBP	18.49 ± 2.18^c^	12.42 ± 0.89^c^	37.36 ± 1.69^c^
FBP	71.32 ± 3.78^a^	16.49.99 ± 0.26^a^	112.36 ± 5.60^a^

a,b,c *Represents the highest figure in the data*.

It had been proven that fermentation could improve the antioxidant activity of barley powder, mainly due to the release of free bioactive compounds caused by biological acidification and microbial enzyme activity (49). A similar result has been reported by Mao et al. (16) for the wheat bran and Chu et al. (50) for the millet bran. Extrusion probably caused the breakdown of phenolic, while fermentation helped to dissolve the phenols as well as improve the extraction efficiency. The increased content of total phenolic contributed to the antioxidant activity and that was consistent with the previous report (51).

## Conclusion

The contents of phenols, β-glucan, protein, and lipid decreased after extrusion. A variety of nutrients and the content of essential amino acids increased after fermentation. The antioxidant capacity of EBP was lower than that of BP, and that of FBP was higher than that of BP, which was consistent with the change of phenolic content. Both Maillard reaction during extrusion, and enzymatic browning during fermentation of barley flour can lead to the darkening of the color, and can effectively optimize the palatability of the barley powder by reducing the viscosity. The RDS content of EBP and FBP were higher than that of BP, which indicated that both extrusion and fermentation could improve the digestibility of barley. The results can be used in the application of fermentation and extrusion in the food industry, especially in the production of high digestibility food such as baby rice paste and feed.

## Data Availability Statement

The original contributions presented in the study are included in the article/supplementary material, further inquiries can be directed to the corresponding author.

## Author Contributions

XX designed the scheme and provided funding acquisition and project administration. JL wrote the main part of the manuscript and participated in the planning and execution of all the experiments. HX assisted in part of the experiment and analyzed it. WT helped with some of the experiments. YZ participated in the design of the experimental scheme. JZ was responsible for writing, reviewing editing, and supervision. All authors read and approved the final manuscript.

## Funding

This work was supported by the Yangzhou key research and development project (modern agriculture) [YZ2020043]; Jiangsu Province agricultural independent innovation project [CX (20) 2036].

## Conflict of Interest

The authors declare that the research was conducted in the absence of any commercial or financial relationships that could be construed as a potential conflict of interest.

## Publisher's Note

All claims expressed in this article are solely those of the authors and do not necessarily represent those of their affiliated organizations, or those of the publisher, the editors and the reviewers. Any product that may be evaluated in this article, or claim that may be made by its manufacturer, is not guaranteed or endorsed by the publisher.
